# Supplementary material for: Pathological Studies on Hantaan Virus-Infected Mice Simulating Severe Hemorrhagic Fever with Renal Syndrome
Source: Viruses. 2022 Oct 13;14(10):2247. doi: 10.3390/v14102247 (PMC9607386; doi:10.3390/v14102247)
Supplement: Supplementary file 1 [file viruses-14-02247-s001.zip › viruses-1877575-supplementary.pdf]

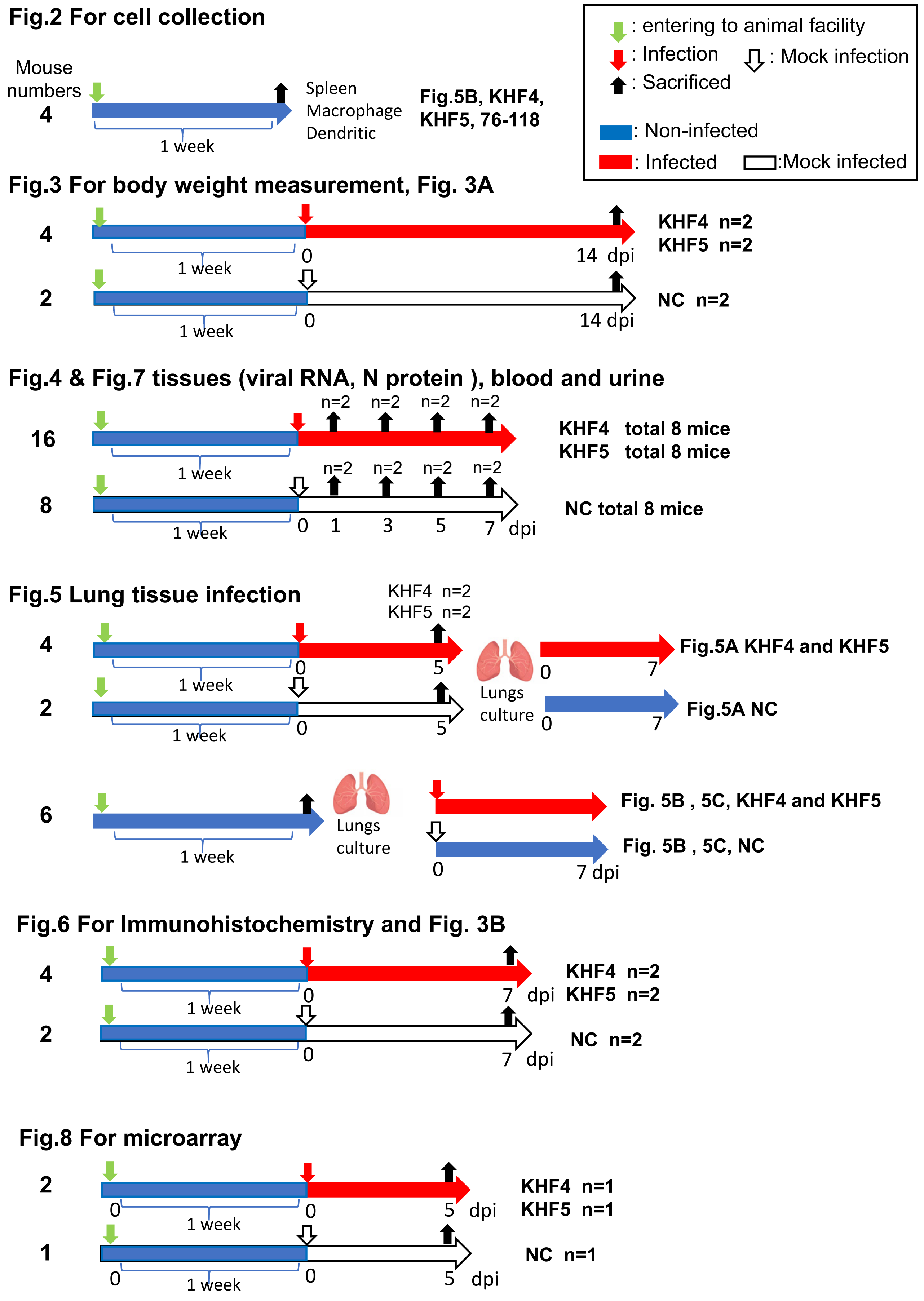

Supplementary Figure S1 Outline of animal experimentation

A total of 55 female BALB/c mice, 4-weeks old were purchased and used in this research.

	N protein /GAPDH	
Mouse ID	Lung	Liver
KHF4 #1	0.13	1.12
KHF4 #2	0.44	1.11
KHF5 #1	1.95	1.73
KHF5 #2	1.67	2.17
NC #1	0.00	0.04
NC #2	0.02	0.05

Supplementary Table S1 Comparison of N protein production in lung and liver
Western blot images of lung and liver shown in Fig. 4B were quantified by Fiji/ImageJ software. Ratio of N protein to GAPDH was estimated in liver and lung tissues.

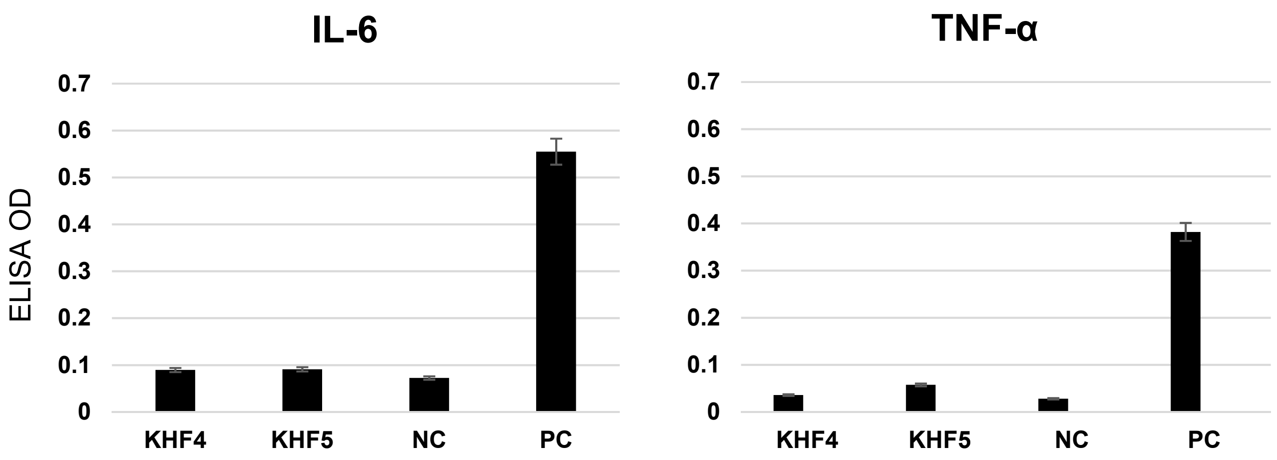

Supplementary Figure S2 Detection of IL-6 and TNF-alpha in sera
Sera were obtained from KHF4, KHF5, and mock infected mice (n=2). Sera were examined at 1:3 dilutions. Positive control IL-6 (333.3 pg/ml) and of TNF-alpha (187.5 pg /mL) were used. All test were performed in duplicate.

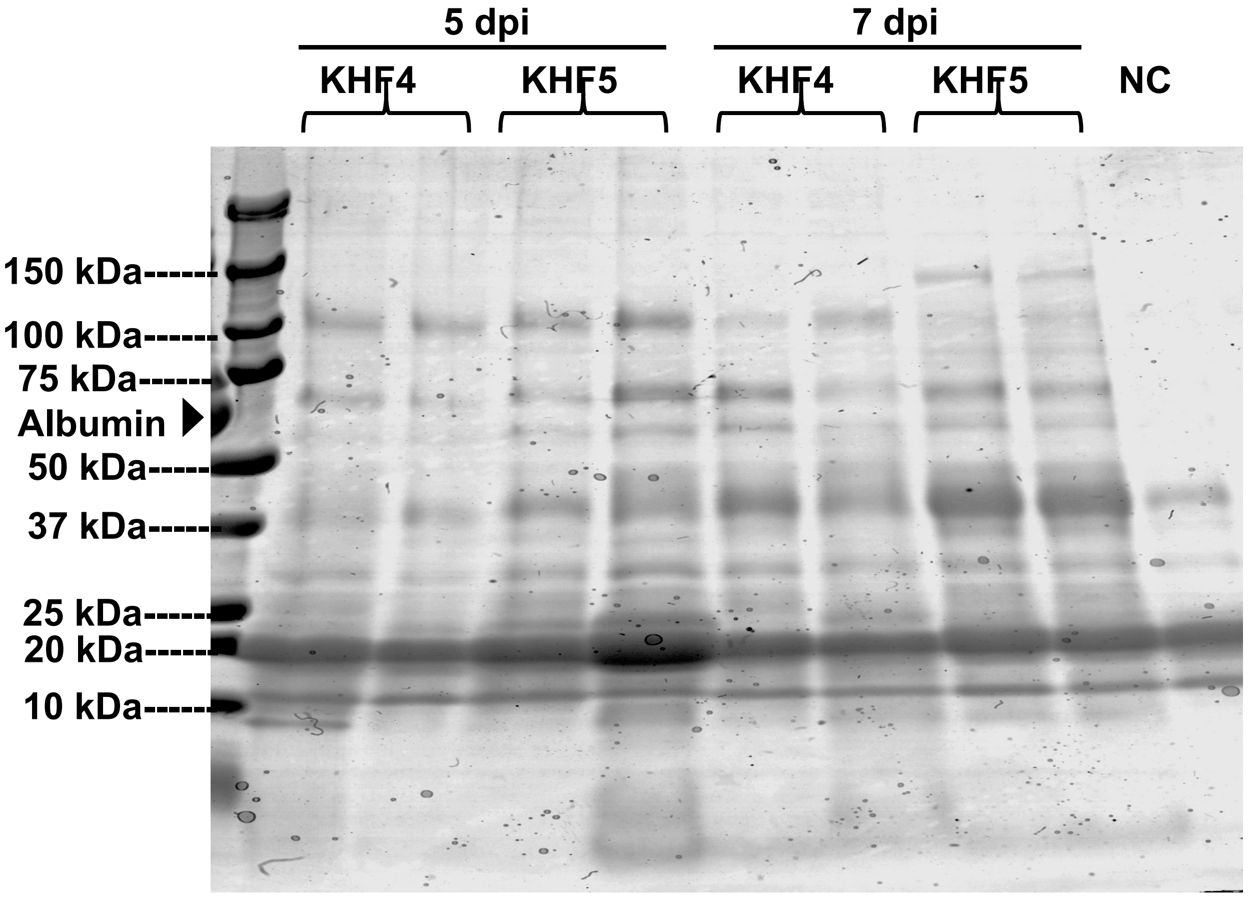

Supplementary Figure S3 Urinary protein analysis by SDS-PAGE

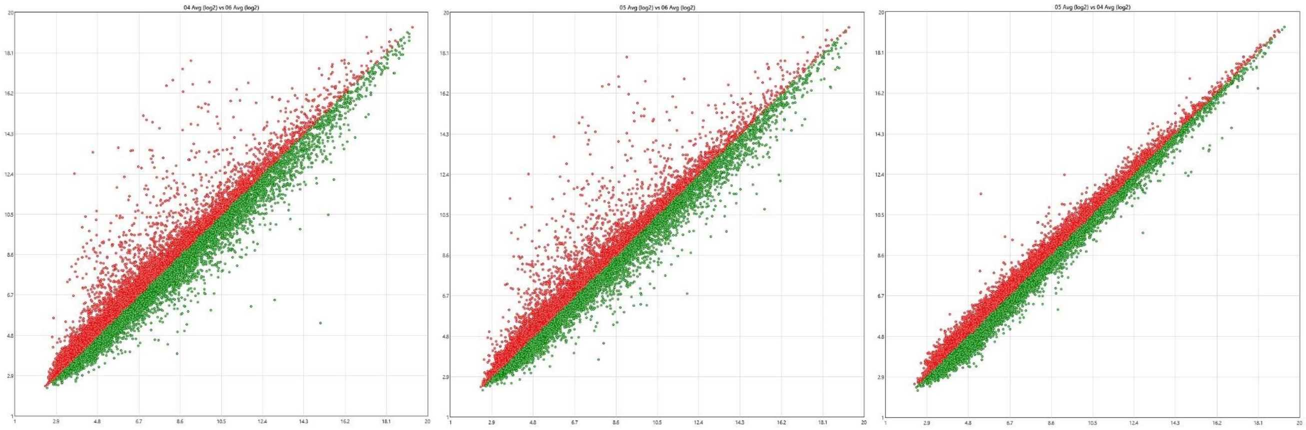

KHF4 VS NC　　KHF5 VS NC　　KHF4 VS KHF5

Supplementary Figure S4 Liver gene expression profiling by microarray
